# Differentially expressed lncRNAs in SOD1^G93A^ mice skeletal muscle: H19, Myhas and Neat1 as potential biomarkers in amyotrophic lateral sclerosis

**DOI:** 10.1098/rsob.240015

**Published:** 2024-10-16

**Authors:** Tresa López-Royo, Laura Moreno-Martínez, Pilar Zaragoza, Alberto García-Redondo, Raquel Manzano, Rosario Osta

**Affiliations:** ^1^ LAGENBIO, Network Center for Biomedical Research in Neurodegenerative Diseases (CIBERNED), Agroalimentary Institute of Aragon (IA2), Institute of Health Research of Aragon (IIS), University of Zaragoza, Calle Miguel Servet 177, 50013 Zaragoza, Spain; ^2^ Neurology Department, ALS Unit, Hospital 12 de Octubre Health Research Institute (i+12), CIBERER U-723 (Instituto de Salud Carlos III), Avenida Córdoba, s/n, 28041 Madrid, Spain

**Keywords:** long-non-coding RNAs (lncRNAs), amyotrophic lateral sclerosis, biomarkers, diagnosis, prognosis

## Abstract

Amyotrophic lateral sclerosis (ALS) is a devastating neuromuscular disease characterized by progressive motor function and muscle mass loss. Despite extensive research in the field, the underlying causes of ALS remain incompletely understood, contributing to the absence of specific diagnostic and prognostic biomarkers and effective therapies. This study investigates the expression of long-non-coding RNAs (lncRNAs) in skeletal muscle as a potential source of biomarkers and therapeutic targets for the disease. The expression profiles of 12 lncRNAs, selected from the literature, were evaluated across different disease stages in tissue and muscle biopsies from the SOD1^G93A^ transgenic mouse model of ALS. Nine out of the 12 lncRNAs were differentially expressed, with Pvt1, H19 and Neat1 showing notable increases in the symptomatic stages of the disease, and suggesting their potential as candidate biomarkers to support diagnosis and key players in muscle pathophysiology in ALS. Furthermore, the progression of Myhas and H19 RNA levels across disease stages correlated with longevity in the SOD1^G93A^ animal model, effectively discriminating between long- and short-term survival individuals, thereby highlighting their potential as prognostic indicators. These findings underscore the involvement of lncRNAs, especially H19 and Myhas, in ALS pathophysiology, offering novel insights for diagnostic, prognostic and therapeutic targets.

## 1. Introduction

Amyotrophic lateral sclerosis (ALS) is a neurodegenerative disease (NDD) characterized by the progressive and selective loss of motor neurons, resulting in muscle weakness, atrophy and dysfunction at the neuromotor junction. It is among the most common NDDs, with an annual incidence of 2.1 to 3.8 per 100 000 people in Europe [1]. Despite its relatively low incidence, ALS is a devastating disease of unknown aetiology and with no cure. It has a typical life expectancy of less than 5 years after symptom onset, although disease progression is highly variable among patients.

Finding biomarkers for early and specific diagnosis of ALS, together with prognostic indicators of disease progression rate, is essential to extend the lifespan and improve the quality of life of these patients. Biomarkers are also of particular interest in elucidating ALS aetiology and pathogenic mechanisms, as well as unravelling potential intervention targets.

Recently, skeletal muscle has gained significant attention as a source of candidate biomarkers in ALS due to the mounting evidence of its active contribution to disease pathology and its accessibility as compared to central nervous system (CNS) sample collection [2]. On this basis, differences in the concentration of prognostic biomarkers identified in hindlimb muscles from the SOD1^G93A^ mouse model of ALS have been found to correlate with disease progression in patients’ blood samples [3,4].

Multiple cellular processes have been associated with ALS pathophysiology in muscle, including oxidative stress, mitochondrial and muscle metabolism dysfunction, protein aggregation, disrupted proteostasis, RNA processing abnormalities, impaired muscle regeneration and dysfunctional stem cell activity [2]. Nevertheless, recent research has highlighted the crucial role of RNA metabolism in the development of ALS. This notion is supported by the identification of mutations in genes responsible for RNA transcription or turnover (such as *SOD1*, *C9orf72*, *TARDBP* and *FUS*) in a significant number of familial ALS cases. Additionally, certain RNA-binding proteins, including hnRNPA2B1, MATR3, TAF15 and TIA1, have been implicated in ALS pathology [5,6]. Therefore, molecules involved in the regulation of RNA metabolism, such as long-non-coding RNAs (lncRNAs), could represent promising candidates for biomarkers studies in ALS.

LncRNAs are RNA molecules of more than 200 nucleotides in length that are not translated into protein. They share similarities with protein-coding messenger RNAs (mRNAs) in terms of sequence length and transcriptional and post-transcriptional behaviour [7]. However, lncRNAs do not typically encode proteins and instead have distinct cellular functions. The precise roles of many lncRNAs are still under investigation, but it is well documented that they participate in various gene regulation pathways at the epigenetic, transcriptional, post-transcriptional, translational and post-translational levels. These pathways include splicing, mRNA turnover and translation, as well as methylation or signalling pathways in response to DNA damage and stress [8,9]. Consequently, lncRNAs influence critical cellular functions such as proteostasis, autophagy, apoptosis, inflammation, cell differentiation or cell cycle regulation and alteration of their expression prompt to numerous diseases [10–17].

Another major distinction between lncRNAs and mRNAs is their greater tissue specificity in terms of expression patterns and functions [7,18]. In fact, lncRNAs have been recently shown to exhibit sex- and disease-specific expression patterns, which highlights their potential as biomarkers and provides a rationale to target them clinically [19–24].

Importantly, several studies have reported potential alterations in lncRNA expression in ALS patients and mouse models by performing both transcriptional and bioinformatic studies [25–34]. However, most of them are cross-sectional studies focused on the CNS or peripheral blood mononuclear cells.

This work is the first one addressing the differential expression of lncRNAs in ALS muscle and, in addition, integrating a longitudinal dimension in the study. In this line, it reveals differences in lncRNA expression in SOD1^G93A^ muscles at different disease stages and correlation of H19 and Myhas with the lifespan of these animals.

## 2. Material and methods

### 2.1. Animals

Transgenic B6SJL-Tg SOD1^G93A^ mice were purchased from the Jackson Laboratory (Bar Harbor, ME, USA) and used as a suitable ALS disease model. Experimental hemizygous transgenic mice were obtained by breeding hemizygous SOD1^G93A^ males with C57BL/6 J × SJL/J F1 hybrid females (B6SJLF1) purchased from Janvier Labs (SaintBerthevin Cedex, France). Wild-type (WT) littermate mice were used as controls. The offspring were identified by PCR assay from tail DNA as described in the Jackson Laboratory protocol.

Mice were hosted at the animal facilities in Centro de Investigación Biomédica de Aragón in a pathogen-free environment and under a standard light/dark (12 : 12) cycle. Food and water were provided ad libitum.

All experimental procedures were approved by the Ethics Committee for Animal Experiments from the University of Zaragoza and were registered with the following code numbers: PI08/19 and PI52/21. The humane endpoint (HEP) for these mice was defined as the loss of righting reflex as shown by a failure to right after laying a mouse on its side for 30 s [35].

### 2.2. Sample collection

Quadriceps samples were isolated from 24 mice at three different stages: 60, 100 and 120 days of postnatal life (P60, P100 and P120), as they correspond to the presymptomatic, symptomatic and late symptomatic stages, respectively (*n* = 12 transgenic SOD1^G93A^ mice and 12 WT mice, balanced males and females, per stage). Muscle samples were subsequently frozen in dry ice and stored at −80°C until processed.

**Figure 1.**
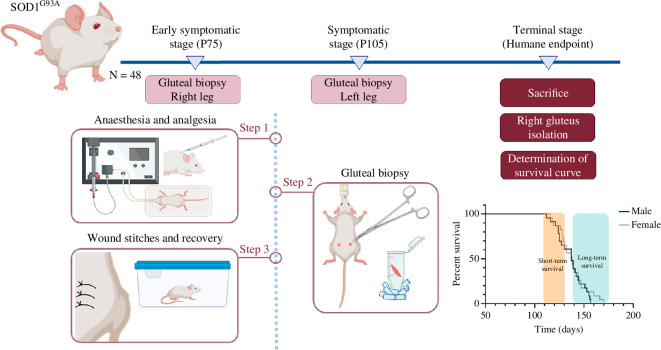
Serial gluteal biopsy procedure. Superficial gluteus biopsies were obtained from 48 sex-balanced SOD1^G93A^ mice at three different time points: early symptomatic stage (P75), symptomatic stage (P105) and terminal stage (HEP). Before the procedure, mice were subjected to anaesthesia and analgesia. Each biopsy was collected from alternating hind limbs starting from the left side. For survival analysis, the age at which the animals reached the HEP was considered as the point of death. Once all animals reached their HEP, survival curves were plotted and animals were divided according to their life expectancy as follows. Animals with a lifespan lower than 130 days were considered as *short-term survival* individuals while those with lifespan higher than 140 days were classified as *long-term survival*. Figure illustrated with Biorender.

To study correlations among each lncRNA expression and longevity, serial samples were collected as previously described in our laboratory by Calvo *et al*. [3]. Briefly, three biopsies of the gluteus muscles were taken from 48 SOD1^G93A^ mice (*n* = 24 mice per sex). Prior to the procedure, mice were anaesthetized with isoflurane and received a subcutaneous injection of analgesic (meloxicam 0.5 mg kg^−1^). Biopsies were collected at 75 days (early symptomatic stage), 105 days (symptomatic stage) and HEP (terminal stage), from alternating hind limbs in consecutive biopsies (figure 1). The age at which animals reached the HEP (described above) was considered as death for survival analysis.

### 2.3. RNA extraction

For RNA extraction, skeletal muscle samples were homogenized by Tissue Lyser LT (Qiagen; Hilden, Germany). Total RNA from quadriceps was isolated using a Direct-zol^TM^ RNA MiniPrep Kit (Zymo Research; Irvine, CA, USA), according to the manufacturer’s protocol. For biopsies from the gluteus muscle, the RNeasy Micro Kit from Qiagen, specific for RNA extraction from a minimal amount of starting tissue, was used.

### 2.4. Real-time polymerase chain reaction

For quantification of lncRNA expression, RNA was retrotranscribed with a high capacity cDNA reverse transcription kit (Thermo Fisher Scientific; Waltham, MA, USA). Quantitative reverse transcription polymerase chain reaction (qRT-PCR) was performed from diluted cDNA in triplicates using a Quant Studio^TM^ 3 real-time PCR instrument (Thermo Fisher Scientific). Custom self-designed Syber Green Primers (Thermo Fisher Scientific) were used in this study at 300 nM concentration (see electronic supplementary material, table S1).

The relative gene expression was determined by the 2^−∆∆CT^ method, using *Gapdh* and *Actb* as housekeeping genes for normalization [36]. For expression studies, mean values from the control group (WT animals) were used to calculate ∆∆CT, while in the biopsy correlation studies, an external reference sample was introduced for comparison between plates, as described in [37].

### 2.5. Statistical analysis

Results are shown as the mean value ± the standard error of the mean (SEM) or standard deviation (SD) as indicated. To establish significant differences between WT and SOD1^G93A^ groups in gene expression studies, a Student’s *t*‐test was performed for each of the lncRNAs analysed. Outliers for each lncRNA and group in expression studies were identified as those with an expression value above the upper limit, or below the lower limit calculated with the following equations [38]:


Upper limit=(75th percentile)+(1.5×(75th percentile)−(25th percentile)),

Lower limit=(25th percentile)−(1.5×(75th percentile)−(25th percentile)).

The correlation of survival with lncRNA levels was assessed using the Pearson correlation coefficient test for normally distributed data and non-parametric Spearman correlation otherwise. In this study, which involved a larger sample size, outliers were identified using Grubbs’ test. This correlation analysis was conducted with two distinct variables. First, from a cross-sectional perspective, the expression levels of lncRNAs (−ΔΔct values) at the early stages of the disease were correlated with survival to identify potential prognostic biomarkers. Second, considering the serial samples and the longitudinal nature of the study, the slope of the lncRNA levels (calculated for each animal using −ΔΔct values at the three different time points) was also correlated with survival. This approach aimed to determine which lncRNAs could either contribute to or reflect muscle damage.

The software used for the statistical analysis was GraphPad Prism version 8.0.1. Differences were considered statistically significant if *p *< 0.05 (*), *p *< 0.01 (**) or *p *< 0.001 (***).

## 3. Results

### 3.1. SOD1^G93A^-induced disease progression modifies skeletal muscle lncRNA expression profile

To identify potential biomarker candidates for ALS, the expression pattern of 12 lncRNAs was evaluated in skeletal muscle samples from SOD1^G93A^ mice at the presymptomatic (P60), symptomatic (P100) and late symptomatic (P120) stages. The SOD1^G93A^ transgenic mouse reproduces numerous features of ALS, such as motor neuron loss, muscle denervation and gradual muscle atrophy and weakening [39–41]. Consequently, this model presents a valuable opportunity to investigate temporal patterns of biomarkers throughout disease progression.

LncRNAs assessed were selected based on the existing literature. Candidates were chosen from those that had been previously described in *in vitro* and *in silico* studies of ALS (such as Malat1 and Neat1) [24,29,34,42–45], as well as those that had been reported to be involved in other neurodegenerative diseases (namely Meg3, Hotair, Gas5, Xist, Snhg1, Snhg16 and Pvt1) [46–49]. Additionally, lncRNAs related to muscle atrophy and other relevant processes in muscle were also included in this study (Pvt1, Myhas, Myoparr and H19) [50,51].

**Figure 2.**
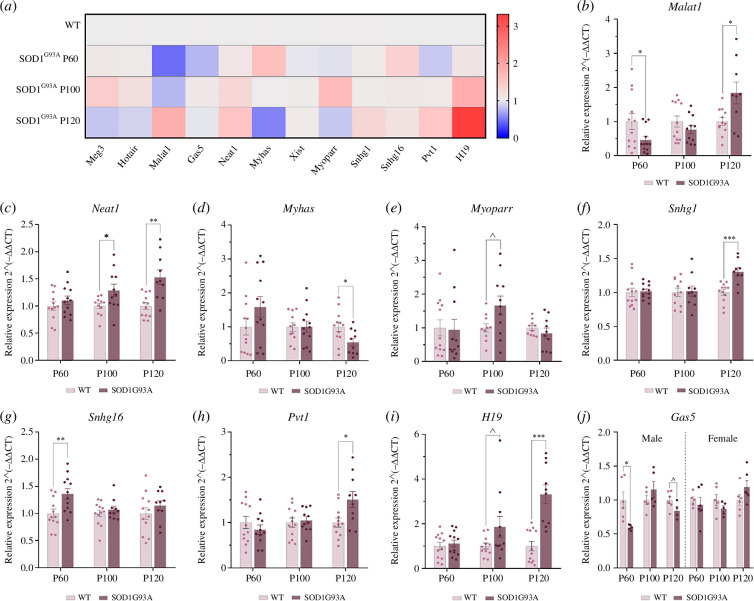
Differential lncRNA expression in quadriceps of control and SOD1^G93A^ mice at different ages. (*a*) Differential lncRNA expression heat map. (*b–j*) Transcription levels of Malat1 (*b*), Neat1 (*c*), Myhas (*d*), Myoparr (*e*), Snhg1 (*f*), Snhg16 (*g*), Pvt1 (*h*), H19 (*i*) and Gas5 (*j*) were assessed in *n* = 24, 60- and 100-day-old mice (balanced in sex and genotype) and *n* = 22, 120-day-old mice (*n* = 4 male SOD1^G93A^, *n* = 6 female SOD1^G93A^, *n* = 6 male WT and *n* = 6 female WT). Expression levels were evaluated by real-time PCR, using the WT mean value for each age as a reference. Data are expressed as mean ± SEM. ^*p *< 0.10, *p < 0.05, **p < 0.01, ***p < 0.001 versus age-matched WT.

Nine of those lncRNAs were differentially expressed throughout disease progression in transgenic SOD1^G93A^ mice skeletal muscle when compared to sex- and age-matched WT controls (figure 2). Among them, eight (Malat1, Neat1, Myhas, Myoparr, Snhg1, Snhg16, Pvt1 and H19) displayed a similar profile in both sexes, whereas Gas5 was shown to be selectively misregulated in males (electronic supplementary material, table S2 and figure S1).

Interestingly, most of the studied lncRNAs, in particular Neat1, Myoparr, Snhg1, Pvt1 and H19, were up-regulated in symptomatic stages of the disease. The presence of impairment during these stages could indicate the potential involvement of these lncRNAs in ALS pathological processes.

On the other hand, lncRNA expression changes during the presymptomatic stage may suggest a role in pathogenesis and/or development of ALS. In this sense, only Gas5 and Snhg16 were differentially expressed at P60, Gas5 being down-regulated in males and Snhg1 up-regulated.

Remarkably, Malat1 and Myhas exhibited an altered expression profile that shifted over the disease course. This pattern proved opposite for the two lncRNAs, with Malat1 being diminished at P60 and P100 in SOD1^G93A^ and up-regulated at P120 and Myhas being increased at P60 in females and decreased at P120 in the SOD1^G93A^ mice. This shift might suggest involvement in biological processes with a dual role in ALS disease.

### 3.2. LncRNAs H19 and Myhas in skeletal muscle can forecast lifespan in SOD1^G93A^ mice

To assess the potential role in disease progression and their value as prognostic biomarkers, we analysed the expression of all previously studied lncRNAs with an altered expression profile in skeletal muscle biopsies of *n* = 48 sex-balanced SOD1^G93A^ mice. For this purpose, gluteal biopsies were collected at different disease stages from the same individual: early symptomatic, symptomatic and HEP, as shown in figure 1. Lifespan of these animals was also recorded, establishing a classification into *short-term* (<130 days) and *long-term* (>140 days) *survival* animals (electronic supplementary material, figure S2).

Four lncRNAs were selected from this analysis for correlating with longevity over time (H19 and Myhas) or at a particular timepoint (Myhas, Snhg1 and Gas5). We hypothesized that those lncRNAs correlated with longevity would be more likely to be involved in major ALS pathological processes.

**Figure 3.**
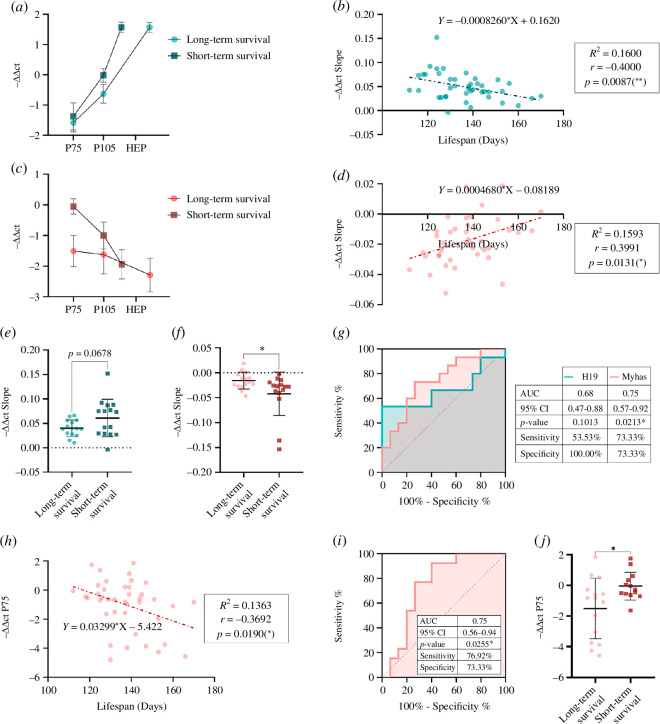
LncRNAs in skeletal muscle can forecast SOD1^G93A^ mice lifespan. (*a,c*) Serial values of −ΔΔct for lncRNAs H19 (*a*) and Myhas (*c*) in *long-* and *short-term survival* SOD1^G93A^ mice. Results are plotted as mean ± SEM at 75 days, 105 days and HEP (123 and 151 days for *short-* and *long-term survival* animals, respectively). (*b,d*) Correlation of −ΔΔct slope with life expectancy for H19 (*b*) and Myhas (*d*). (*e,f*) Statistical differences in −ΔΔct slope between *short- and long-term survival* animals for H19 (*e*) and Myhas (*f*). Data are represented as mean ± SD; *p < 0.05. (*g*) ROC curve for H19 and Myhas slopes. (*h*) Correlation of Myhas −ΔΔct with life expectancy at P75. (*i*) ROC curve for Myhas −ΔΔct at P75. (*j*) Statistical differences in −ΔΔct at P75 between *short- and long-term survival* animals for Myhas. Data are represented as mean ± SD; *p < 0.05. All data were collected from 48 sex-balanced SOD1^G93A^ mice. ROC curve plots discriminate between the *short- and long-term survival* groups. Animals with a life expectancy greater than 140 days were classified as *long-term survival* animals (average lifespan of 151 days), whereas those with a life expectancy less than 130 days were classified as *short-term survival* animals (average lifespan: 123 days).

Among these four, H19 and Myhas correlations were the most robust (minor *p*-value) and applicable to both males and females. H19 levels were shown to increase throughout the disease course, showing a sharper slope in the *short-term survival* group (figure 3*a*). In fact, a statistically significant negative correlation was observed between −ΔΔct slope and the lifespan of each animal (figure 3*b*; results for separated sexes can be found in electronic supplementary material, figure S3). Taken together results from figures 2*h* and 3*a*,*b* suggest an involvement of H19 in pathological mechanisms occurring alongside disease progression and modifying survival in this animal model.

In contrast, Myhas decreased across disease progression (figure 3*c*). Results shown in figures 2*c* and 3*c* suggest an up-regulation of Myhas levels in SOD1^G93A^ skeletal muscle at the presymptomatic and early symptomatic stages. However, as the disease progresses to the late symptomatic stage, these levels decrease and become lower than those in WT mice. Thereby, SOD1^G93A^ mice with a shorter life expectancy had higher levels of Myhas during the early symptomatic stage and a more pronounced decrease in Myhas skeletal muscle expression throughout the disease as compared to *long-term survivors* (figure 3*c*). Correlation of SOD1^G93A^ lifespan with either Myhas expression at early symptomatic stage (−ΔΔct at P75, figure 3*h* and electronic supplementary material, figure S4; separated sexes) or Myhas progression along the disease (−ΔΔct slope, figure 3*d* and electronic supplementary material, figure S5; separated sexes) was statistically significant.

Furthermore, to determine the robustness of H19 and Myhas as prognostic biomarkers, statistical *t*-tests and ROC curves were performed to see if they were able to discriminate between groups of SOD1^G93A^ animals with short and long survival.

The *t*‐test values for H19 (*p* = 0.0678, figure 3*e*) and Myhas (*p* = 0.0371, figure 3*f*) and ROC curves (figure 3*g*) suggest that the −ΔΔct slopes of these lncRNAs could predict SOD1^G93A^ mice survival range (short versus long). Specifically, for H19, the ROC curve cut-off of 0.0690 discriminates animals with fast versus slow progression with a sensitivity of 53.53% and a specificity of 100% (*p* = 0.1013). For Myhas, this cut-off is set at −0.0239, with a sensitivity and specificity of 73.33% (*p* = 0.0213). Additionally, −ΔΔct of Myhas at P75 was also able to discriminate the speed of progression of the animals (figure 3*i,j*), with a sensitivity of 76.92% and a specificity of 73.33% (*p* = 0.0255, cut-off of −0.5550). These results are particularly robust since both male and female individuals follow the same pattern and highlight the potential of specially Myhas as a candidate prognostic biomarker in ALS.

As for the other lncRNAs studied, correlations were only significant in one sex. In females, Snhg1 levels at the HEP correlated with life expectancy (electronic supplementary material, figure S6*a*,*b*). Notably, this correlation was not significant in males despite the increase of this lncRNA observed in both sexes at the late symptomatic stage in SOD1^G93A^ mice (electronic supplementary material, figure S1I). On the other hand, in males, Gas5 levels at the early symptomatic (P75) and symptomatic (P105) stages showed a positive correlation with longevity (electronic supplementary material, figure S6*c*–*e*). However, the power to discriminate between males with short- and long-term survival proved to be limited (electronic supplementary material, figure S6*b*,*f*–*h*).

Moreover, it is worth mentioning that Malat1 could not be included in this study as the biopsy procedure drastically decreased its expression levels. This occurred at both P75 and P105 time points. We speculate that anaesthetic and/or analgesic drugs might underlie this effect since another anaesthetic, namely propofol, has been already described to restrain the expression of this lncRNA [52–54]. If it is confirmed that Malat1 exhibits high variability in response to different conditions and drugs, this lncRNA is likely not a suitable candidate for a biomarker.

## 4. Discussion

Over the last few decades, there has been an intense effort to identify biomarkers for ALS that can accelerate diagnosis, which currently relies on excluding other possible conditions, and provide insights into patient prognosis as well as new potential therapeutic targets. The search for these biomarkers has involved all sorts of molecules and has primarily focused on tissues such as the brain, spinal cord, cerebrospinal fluid, blood and skeletal muscle. Despite the extensive investigations and the emergence of molecules like neurofilaments entering clinical trials, none of them has been implemented in actual clinical practice yet [55].

The emergence of lncRNAs has opened up new possibilities in the field of biomarker discovery. These molecules show tissue- and sex-dependent expression patterns and functions, which enables the identification of specific disease patterns [19]. Indeed, in recent years lncRNAs have been shown to be impaired and play an important role in several neurodegenerative diseases such as Parkinson’s disease and Alzheimer’s disease or multiple sclerosis [46,56–63]. However, their involvement in ALS is still unclear.

In this work, we first conducted an analysis of 12 lncRNAs’ expression in the muscle of SOD1^G93A^ mouse model of ALS to determine putative dysregulations and to evaluate their potential as disease biomarkers. Skeletal muscle was selected as it is considered a primary target in ALS toxicity [2].

Among the lncRNAs investigated, nine showed statistically significant differences between WT and SOD1^G93A^ animals. Five of them, namely Neat1, Myoparr, Snhg1, Pvt1 and H19, exhibited changes exclusively in the symptomatic stages (P100 and P120); while Gas5 and Snhg16 showed changes in the presymptomatic stage (P60) and became unvariable later on. Of note, Myhas and Malat1 changed their expression pattern throughout the disease course, which could suggest a dual role of these lncRNAs in the disease.

When considering the discovery of potential diagnostic biomarkers, none of the molecules displayed significant changes across all the presymptomatic and symptomatic stages. However, Neat1 and H19 remarkably showed the highest fold change at the symptomatic stages, raising them as good candidates for further study in humans as biomarkers to support and/or confirm ALS diagnosis. Furthermore, Pvt1 also showed a notable increase in the late symptomatic stage of the disease in male subjects, which is particularly relevant given the increasing number of articles highlighting sex differences in ALS [64,65]. This lncRNA might serve as a candidate diagnostic biomarker in men, but also as a potential ALS therapeutic target, as it has been found to be up-regulated in several models of muscle atrophy and impact fundamental processes in skeletal muscle, including mitochondrial respiration and morphology, mito-/autophagy and myofibre size [66]. The need for such biomarkers persists today, given the lengthy diagnostic timeline (of over 10 months on average from symptom onset to definitive diagnosis in ALS cases). The development of a comprehensive panel of clinical tests and biomarkers is crucial to improve the differential diagnosis of other ALS-mimicking conditions and thus speed up the current diagnostic process.

Interestingly, Neat1 is an essential component of paraspeckles [67], intranuclear bodies increased in the spinal cord of sporadic patients and *in vitro* models of ALS motor neurons [30,42]. Paraspeckles are also involved in the regulation of RNA metabolism, the disruption of which is a hallmark of ALS [68,69]. As for the Neat1-specific role in muscle, *in vitro* studies suggest that this lncRNA enhances myoblast proliferation while suppressing differentiation and fusion [70]. Consequently, elevated levels of Neat1 could potentially halter muscle regeneration, which is impaired in the disease [71–73]. The up-regulation of this lncRNA in different forms of the disease (sporadic and familial—associated with mutations in different genes such as FUS and SOD1^G93A^) and in the most affected tissues (motor neurons and skeletal muscle) supports the hypothesis that this lncRNA may be a strong candidate biomarker to aid in diagnosis. Furthermore, a recent study suggests Neat1 as a possible key player in disease progression and genetic modifier of age at onset in ALS [34].

Regarding H19, besides being found markedly elevated in SOD1^G93A^ mice, serial biopsy samples demonstrated that H19 levels increased as the disease progressed, with a faster rate of increase associated to shorter longevity. These findings suggest a link between H19 levels and muscle damage, with H19 acting either as an indicator of this damage or as a potential contributor to the pathogenesis of the disease. Given that muscle damage reflects ALS progression, H19 might be a valuable prognostic biomarker. Additionally, it might serve as a drug response biomarker to monitor the effectiveness of treatments in slowing or alleviating muscle damage in clinical trials.

The role of H19 in adult skeletal muscle is well characterized since in adults this lncRNA is predominantly expressed in this tissue. This lncRNA is described to favour myogenesis and satellite cell-mediated differentiation, enhancing muscle regeneration [74]. Besides, it has been associated with glucose metabolism, insulin response and dystrophin stabilization [75]. Indeed, H19 levels in this tissue need to be tightly regulated since recent studies suggest that H19 elevated levels may also lead to skeletal muscle atrophy and fibrosis [76,77]. In light of these findings, H19 may also represent a compelling target for ALS treatment, based on the hypothesis that elevated levels of this lncRNA may contribute to muscle atrophy and skeletal muscle fibrosis, as previously discussed.

In addition to H19, three other lncRNAs (Snhg1, Gas5 and Myhas) correlated with longevity in SOD1^G93A^ animals.

Snhg1 expression was found to be elevated in SOD1^G93A^ mice, with a negative correlation with longevity during the terminal phase of the disease in females. The specific role of this lncRNA in skeletal muscle has not been investigated, although current evidence suggests a potential role in myocyte regeneration [78–80]. Our results indicate that Snhg1 may act as a late agent in disease progression or as an insufficient compensatory mechanism for muscle damage active in the late stages of ALS.

In males, animals with a longer lifespan showed higher expression of Gas5 at the early phases of the disease. Since this correlation occurs early in disease course, Gas5 may serve as a good prognostic indicator in males. Consistent with this finding, Gas5 was previously observed to be decreased in presymptomatic SOD1^G93A^ male mice as compared with their age- and sex-matched WT littermates. This initial down-regulation observed might be relevant for disease onset and development in males, and could presumably indicate a protective role of Gas5 in muscle. Gas5 has been linked to cell cycle regulation, apoptosis and inflammation, but its role remains controversial since opposite effects have been reported depending on context and tissue [81–86]. In line with the putative protective role of Gas5 in ALS skeletal muscle, it has been shown that Gas5 is related to the inactivation of NRLP3 inflammasome in cardiac muscle [87,88]. Therefore, Gas5 down-regulation in males could be related to a hyper-activation of the inflammasome in early stages, which has been previously reported in the skeletal muscle of this animal model and patients with ALS [89], in this sex. Interestingly, these differences were observed exclusively in males, who exhibit earlier pathology than females in this animal model.

Finally, Myhas levels showed a decreasing pattern throughout the disease, with an initial increase relative to the WT controls during the presymptomatic stage, followed by a subsequent decline in the late stage. Notably, animals with shorter life expectancy exhibited a greater increase in Myhas expression during the early symptomatic stage, resulting in a more abrupt decline. Previous research has evidenced that this lncRNA promotes myogenesis and maintains the fast-twitch phenotype in muscle fibres [90]. Accordingly, our results suggest that during the presymptomatic stage, increased levels of Myhas may stimulate satellite cell division, differentiation and fusion into myotubes, thereby maintaining the fast-twitch fibre phenotype, which is more susceptible to degeneration. This hypothesis could explain why animals with a greater increase in this lncRNA during the early symptomatic stage have a shorter life expectancy. Once the compensatory mechanisms aimed at increasing satellite cell proliferation are exhausted, Myhas levels would decline, in turn favouring the shift towards a slower muscle fibre phenotype. This idea would be consistent with the observation that, as ALS progresses, there is a shift towards differentiation into slow twitch muscle fibres (which show greater resistance to denervation) [91]. Furthermore, of all the lncRNAs studied, Myhas demonstrated the greatest potential to discriminate between *short-* and *long-term survival* groups. These results not only suggest a potential role for Myhas in the pathophysiology of muscle in ALS, but also propose it as a promising prognostic biomarker.

## 5. Conclusion

This article points out lncRNAs as valuable players in the pathophysiology of ALS muscle. Further research will be needed to unravel their detrimental or protective role in the disease, as well as the putative pathways involved. Moreover, the reported Myhas and H19 correlation with longevity opens the path to further evaluate their potential as prognostic and drug response biomarkers in human patients and be used as promising therapeutic targets to delay disease progression and prolong lifespan.

## Data Availability

All data generated or analysed during this study are included in this published article and its electronic supplementary material. Further information is available from the corresponding author on reasonable request. The content is solely the authors’ responsibility and does not necessarily represent the official views of the University of Zaragoza and the Hospital 12 de Octubre Health Research Institute. Supplementary material is available online [92].
